# In the Eye of the Storm: The Role of the Pharmacist in Medication Safety during the COVID-19 Pandemic at an Urban Teaching Hospital

**DOI:** 10.3390/pharmacy8040225

**Published:** 2020-11-21

**Authors:** Abir O. Kanaan, Karyn M. Sullivan, Sheila M. Seed, Nathan A. Cookson, Linda M. Spooner, George M. Abraham

**Affiliations:** 1 School of Pharmacy Worcester/Manchester, MCPHS University, Worcester, MA 01608, USA; abir.kanaan@mcphs.edu (A.O.K.); karyn.sullivan@mcphs.edu (K.M.S.); linda.spooner@mcphs.edu (L.M.S.); 2 Saint Vincent Hospital, Worcester, MA 01608, USA; Nathan.Cookson@stvincenthospital.com (N.A.C.); george.abraham@stvincenthospital.com (G.M.A.)

**Keywords:** pharmacy, pharmacist, medication safety, COVID-19

## Abstract

The severe acute respiratory syndrome coronavirus 2 (SARS-CoV-2), the virus responsible for the coronavirus disease (COVID-19) pandemic, has challenged practitioners with complex clinical scenarios as well as conflicting and scarce data to support treatment strategies. The pandemic has also placed strains on institutions due to drug shortages, alterations in medication use processes, economic losses, and staff exposure to the virus. This article provides pharmacist-led suggestions and strategies to various case questions, describing some of the challenges faced by practitioners at an urban teaching hospital during the COVID-19 pandemic. The strategies suggested can be explored at other institutions.

## 1. Introduction

The novelty of the severe acute respiratory syndrome coronavirus 2 (SARS-CoV-2), the virus responsible for the coronavirus disease (COVID-19) pandemic, has challenged standard practices in healthcare and given rise to questions, with limited evidence and resources to provide answers. In this commentary, we provide answers to questions we received at our institution, a teaching hospital, with the goal of offering guidance which health practitioners could explore to inform their practice.

## 2. Materials and Methods 

A team of clinical pharmacists and an infectious disease physician specialist compiled challenges that were encountered at a 283-bed, inner-city hospital during the height of the COVID-19 pandemic from March 2020 to August 2020. The clinical pharmacists completed general and/or specialized residency training or graduate studies. Their areas of practice include infectious diseases, internal medicine, cardiology, medication safety, travel medicine, and public health. Chosen for this commentary were challenges that required a change in the role of the pharmacist with respect to medication safety practices, the management of drug shortages, and the determination of clinical scenarios that required pharmacist-led suggestions regarding data to support treatment options. The authors designed each case based on questions they received or experiences they encountered, collaborated on providing answers, and assembled information to develop this commentary.

## 3. Results

### 3.1. Challenges with Answers and Practice Learning Points

#### 3.1.1. Contributions of Pharmacy and Pharmacy Extenders (Students, Residents, and Fellows) to Monitoring of Medications Used to Treat Patients with COVID-19

Case: Practitioners at your institution are bombarded with information about potential treatment options for COVID-19, from listserv postings to daily online journal updates to the evening news. The pharmacy staff receive multiple inquiries per day while attending to their operational and clinical responsibilities. How can healthcare professionals stay abreast of the rapidly changing information regarding COVID-19 pharmacotherapy, and how should the evidence base for the monitoring and assessment of these pharmacotherapeutic regimens be incorporated into daily pharmacy operations to ensure medication and patient safety? 

Answer: The vast amount of dynamic pharmacotherapeutic information made available throughout the pandemic has been unprecedented. This, along with the presence of misinformation in the professional and lay press, is extremely challenging to keep pace with, especially with all of the novel tasks that have arisen within institutions during this time. Pharmacists and pharmacy extenders (e.g., pharmacy students, residents, and fellows) are positioned in an ideal role to assist with the review, analysis, and assessment of this immense amount of data. 

During their experiential education, pharmacy students have the knowledge and training that would allow them to conduct literature searches, assimilate information, and make recommendations that are evidence-based. Their work in courses such as pharmacotherapy, pharmacology, medicinal chemistry, and pharmacokinetics provides them with the knowledge, skills, and attitude to contribute to patient care through various activities, including dissemination of drug information. At our institution, pharmacy students provided weekly written updates since late January 2020, when information about the management of COVID-19 was emerging from various parts of the world. As time passed, these updates became more frequent and included state-specific information on the number of COVID-19 cases and deaths, reviews of published data, and research into topics requested by practitioners. Moreover, pharmacy students assimilated information from numerous listservs, continuing education programs and webinars, and daily emails from professional pharmacy organizations, such as the American Society of Health-System Pharmacists, the American Pharmacists Association, and the American College of Clinical Pharmacy. Students’ work was reviewed by a clinical pharmacy preceptor prior to dissemination to confirm accuracy and completeness. These updates continued thrice weekly, were provided to prescribers and infection control staff via electronic communication, and discussed at routine teleconferences. Pharmacy students at our institution play an extremely helpful role in supporting the department of pharmacy by acquiring and summarizing rapidly changing information regarding treatments for COVID-19.

Pharmacy residents and fellows are postgraduate trainees and licensed pharmacists who also play a significant role in the management of patients with COVID-19 by providing assistance with a number of tasks that support clinical, operational, safety, and quality initiatives. At our institution, pharmacy medication safety fellows created and implemented disposable extravasation kits for patient care floors, along with an information sheet detailing remdesivir-associated infiltration management. They also coordinated an increased number of daily pharmacy staff huddles to provide information about treatment protocols, medication shortages, and adverse event monitoring. Written summaries from these huddles were also distributed to the entire pharmacy staff to ensure timely and transparent communication. The fellows were also tasked with conducting prospective and retrospective assessments of potential trends identified in our inpatients that have been noted nationally, such as hypercoagulability. The purpose of such activities was to present emerging data which might impact prescribing and monitoring practices. The additional training of pharmacy residents and fellows is invaluable and provides additional support during a pandemic. 

Furthermore, over the past few months, treatment options for patients diagnosed with COVID-19 have progressed at a rapid pace. Due to the large and sometimes conflicting amount of information available about potential treatment options, a multidisciplinary approach to assessment of proper patient management and monitoring is crucial to optimize outcomes and safety. At our institution, a multidisciplinary team including pharmacists developed protocols to assess patient eligibility and to provide appropriate monitoring for specific COVID-19 treatments. An example of this is illustrated with remdesivir, discussed further below. All protocols were reviewed and discussed with the clinical teams caring for these patients, including the Divisions of Infectious Diseases and Geographic Medicine as well as Pulmonary and Critical Care Medicine. Pharmacy staff reviewed these criteria during daily huddles to ensure proper operationalization and implementation. 

The availability of remdesivir through an Emergency Use Authorization (EUA) has permitted expanded access for management of patients with COVID-19 and prompted additional tasks required by the pharmacy department [1]. Our clinical pharmacists assisted in the procurement, inventory, and patient assessment and monitoring as well as documentation of the use of this drug. This was initiated following a request from the Director of the Division of Infectious Diseases in order to provide support to the medical staff. The collaboration between clinical pharmacists and infectious disease physicians was a key component in optimizing the use of remdesivir. Daily emails from the clinical pharmacist permitted communication of the status of each patient receiving the drug, including patient demographic information, number of days of treatment completed, laboratory data, adverse effects, and recommendations for continuation of therapy. These efforts prevented remdesivir waste, despite the treatment of over 60 patients. In addition, clinical pharmacists collaborated with prescribers to ensure all laboratory parameters were ordered appropriately and that communication was sent at the end of each day to share pertinent information, including doses given. This clinical monitoring service continued seven days per week, which allowed continuity of care. Lastly, clinical pharmacists provided multiple nurse education sessions to ensure the proper administration of remdesivir. Adverse effects were monitored and reported internally and externally, and documentation was reviewed as part of a more formal prospective drug utilization evaluation process. 

Practice/Learning Point: Optimizing use of pharmacy staff and pharmacy extenders is critical for provision of high-quality care in the rapidly changing management of patients with COVID-19. Pharmacy students, residents, fellows, and staff provide great value to other practitioners during a pandemic as they are well-versed with efficacy and safety considerations of drugs used to manage patients with COVID-19. They are also instrumental in evaluating published literature to determine statistical significance and clinical relevance. 

#### 3.1.2. Implementation of the RED Zone as a Measure to Prevent Errors with Laboratory Reporting—Lessons from the Pharmacy Department

Case: One month into the management of COVID-19, about 320 laboratory tests have been performed to detect the presence of the infection in patients treated at our institution. Results from one particular patient are unexpected. The test is positive for COVID-19 but the clinical presentation does not match the findings. What should be done in response to this situation to optimize accurate reporting and patient safety? 

Answer: The decision to treat patients according to unexpected laboratory findings has serious implications, including potentially administering unnecessary medications and an extended hospital stay. Therefore, unexpected positive results should be investigated further. Similarly, false negative results might have devastating implications, as patients would be cleared to go back into the community with high risk of transmission of infection if social distancing is not followed. When an unexpected positive result was reported at our institution, the laboratory reporting process was examined and a review of all reported COVID-19 test results was conducted, revealing five (1.6%) negative results that had been erroneously reported as positive. No false negatives were detected. This led to a root cause analysis involving our risk department, the laboratory supervisor, infection prevention and the Director of the Division of Infectious Diseases to uncover how the erroneous results were reported out. The root cause of the error was determined to be the laboratory reporting process. Specifically, COVID-19 testing was performed outside of the institution and results were received via a digital incoming fax system, discrete from the usual process of the results being input through an electronic interface (automated). A laboratory technician then manually entered the results into our laboratory electronic information system. Manual input of results is not the usual practice for technicians at our institution and was being performed in addition to several other general laboratory tasks. The technicians had been multitasking, distracted, and keyed incorrect results into the system.

Error reduction strategies must be considered once the root cause has been identified. Automation and computerization are among the highest level strategies to consider but were not an option at our institution since the results were being received from an outside laboratory [2]. We recognized that the strategy had to focus on limiting distractions during input of test results; therefore, the RED Zone rule that had been implemented in the pharmacy department many years ago was considered. RED Zone stands for ‘Reduce and Eliminate Distractions’ and is based on the “sterile cockpit” rule from the commercial aviation industry that forbids nonessential flight crew activity and conversation during taxiing, takeoff, and landing [3,4]. Red tape outlines the area used by pharmacists to verify the accuracy of products prepared for dispensing. Pharmacy storage shelves have also been designated as RED Zone areas. Interruptions and talking are not allowed in these zones to permit full focus on the medication-related accuracy steps at hand. No-interruption areas, such as the RED Zone, have been reported in the literature as being effective for reducing distractions and errors when performing critical tasks [5,6,7,8]. 

As a result, a RED Zone was implemented in the laboratory area where technicians input COVID-19 test results into the electronic information system. The sole responsibility of the technician within the RED Zone is to input the test results. Interruptions are not permitted. Rule-based error reduction approaches are low-leverage strategies because they focus on improving human performance [2]. Due to this event, random reviewing of COVID-19 tests has continued and zero reporting errors have been detected among the over 2000 tests performed since we implemented the RED Zone in the laboratory.

Practice/Learning Point: It is important to question unexpected test results even in the midst of a pandemic. Identifying the specific process responsible for the errors made the reduction strategy simple. Although the RED Zone has been shown to decrease errors when performing medication-related tasks, the idea was applied successfully in the laboratory setting to minimize distractions when inputting laboratory data. Learning from existing safety methods applied by the pharmacy department supported the implementation of a strategy in another setting to eliminate errors in data reporting. 

#### 3.1.3. Revising Medication Use Processes to Address Challenges Raised by the COVID-19 Pandemic

Case: A pharmacy technician noticed a patient’s own medication in the medication room when refilling the automated dispensing cabinet. Although the medication was not labeled by the inpatient pharmacy, an information sheet from the outpatient pharmacy was attached. The pharmacy technician gives the medication to the pharmacist. Further investigations suggests that the medication belongs to a patient who is a person under investigation (PUI) for COVID-19. The pharmacist asks the manager at our institution about the need to update pharmacy policies and procedures to account for changes in medication use processes prompted by the pandemic as well as minimizing staff exposure to COVID-19. What processes can be implemented or revised to address the pharmacist’s suggestions?

Answer: In addition to updated laboratory error prevention strategies at our institution, COVID-19 prompted health systems to evaluate many existing medication use processes. Engineering controls which isolate workers from a hazard may be more effective than administrative controls, which change the way workers perform tasks [9]. However, due to the cost and time needed to implement engineering controls, administrative controls and personal protective equipment (PPE) are often employed first. From modified staffing models and work force reductions to bulk product dispensing and medication returns from precaution rooms, healthcare workers and hospital staff made changes to work flow in order to care for patients with COVID-19. Some of these work flow changes may revert back to usual operations (or a new normal) once the crisis is over, but other alterations should persist, permanently affecting the medication use process. 

To start, potentially contaminated medications need to be identified and handled in a way that reduces staff exposure. One approach is to bag medications for transport by using special warning bags to alert staff to possible contamination. Our institution chose to use transparent biohazard bags to transport potentially contaminated items. When a patient’s own medication was removed from a precaution room, a nurse placed the item in a biohazard bag to let the pharmacy technician who brought it back to the pharmacy or the pharmacist who received the medication know that the product should be handled according to our precautions protocol. Limiting access to that medication and not storing it in a medication room is another strategy. Staff should also be encouraged to perform hand hygiene at every opportunity, especially before and after handling patients’ own medications. 

Another important process that needed change pertained to processing items returned to the pharmacy. The first return item addressed was “patients’ own medications”. The pandemic presented an opportunity to reinforce education to nurses that home medications need to be delivered to the pharmacy either by a nurse or by a pharmacy technician because these medications are not allowed to be put through the pneumatic tube system. The common reason for requiring medications to be delivered to the pharmacy is to prevent loss or damage; however, COVID-19 offered an opportunity to evaluate how potentially contaminated patient medications were processed. It was decided that “patients’ own medications” for patients with COVID-19 or PUIs that were planned to be used during the hospitalization would be stored in the patient room after being verified and labeled by a pharmacist. This was to avoid cross-contamination by bringing the medication in and out of an isolation room. For medications that were not to be used but could not be returned home, the pharmacy worked with nurses to identify “patients’ own medications” from patients with COVID-19 or PUIs for storage in the pharmacy; nurses verbally alerted the pharmacy staff to possibly contaminated medication packaging/containers when the medication was dropped off at the pharmacy and brought them down in a sealed, transparent bag. Pharmacy staff were expected to perform appropriate hand hygiene when receiving and handling these medications, which included washing or disinfecting hands with an alcohol-based hand sanitizer, disinfecting the transport bag with alcohol, donning nonsterile gloves before handling the contents of the sealed bag if needed, removing gloves after resealing the bag, and washing or disinfecting hands immediately after removing gloves. Staff were also encouraged to wear gloves while handling the transport bags, regardless of whether manipulation of the bagged contents was required. The Infection Control and Prevention team and nursing leadership reviewed these changes and were helpful in educating nurses on appropriate bagging and transport.

In addition, rapid sequence intubation kits were also changed from a hard case to a sealed, transparent bag to facilitate disinfection. Code cart processing was challenging due to potentially wasting all of the medications that may have been exposed to SARS CoV-2. Pharmacy, nursing, infection prevention, and sterile processing teams worked together to develop a method to identify code carts that were used in patients with COVID-19 or PUIs. The process was updated so that the cart itself would remain outside the patient room, the medication tray would go into the room, and any supplies from the tray would be passed on into the room as required. Any unused medications would be wiped down with an alcohol wipe while still in the patient room and then placed into a clean, plastic, biohazard bag before being returned to pharmacy. The pharmacy sequestered any returned medications for ten days before returning the drugs to working stock. 

To manage our inventory, the pharmacy chose to publish a list of drugs used in the treatment of coronavirus patients, known as a COVID-19 drug inventory. Several resources were used to determine drug availability as well as drug shortages, including available alternatives. The list was sent to administration and department leaders daily during the peak of the pandemic response, and then twice weekly once the surge subsided. The first iteration of the list accounted for medications that were theorized to be useful against the novel coronavirus and included inhalers used in place of nebulizers. A color coding system was also utilized to let prescribers know not only the inventory level but also how readily available each line item was: green meant no issues ordering the drug from our primary supplier; yellow suggested limited availability, allotments, and/or secondary supplier items; red meant unavailable. The list was expanded to include vasopressors, analgesics, sedatives, and paralytics when those products were in shortage. At the height of the surge, there were seventy-five line items for which the pharmacy reported on their availability. The list continued to be updated as new data emerged regarding efficacy outcomes or availability of formulations. For example, rocuronium and fentanyl were added when compounded items from a local 503b pharmacy were available. 

The pandemic revealed several gaps in our operational processes which promoted reassessment of several medication processes. The pharmacy department has now plans in place to handle catastrophic shortages in critical care medications and is working to fully develop secondary and tertiary supply chains. 

Practice/Learning Point: Administrative controls, such as protocol changes and work flow updates, could limit staff exposure to hazards including COVID-19. They are easier to implement than engineering controls, could provide an added level of protection to personal protective equipment, and can be tailored specifically to an institution or department.

#### 3.1.4. Pharmacy Role in Addressing Shortages of Sedatives during the COVID-19 Pandemic

Case: Due to a surge in patients with COVID-19 needing respiratory support, the number of those requiring mechanical ventilation in the Intensive Care Unit (ICU) has peaked. The pharmacy is running short on their supply of propofol and other sedatives. The critical care and surgical teams ask the pharmacist at your institution to recommend other options to conserve sedatives. 

Answer: In a study conducted in twelve New York City hospitals, approximately 12% of patients with COVID-19 required mechanical ventilation, with up to 25% receiving early neuromuscular blockade with paralytic agents [10,11]. In order to keep patients comfortably sedated while intubated, hospitals widely use general anesthetics, such as propofol and paralytics [10]. In areas that experienced surges of patients with COVID-19, elective surgeries were suspended, but as the number of these patients became more manageable, elective surgeries resumed. With an increase in elective surgeries while continuing to care for those patients requiring respiratory assistance with mechanical ventilation in the ICU, shortages and conservation of anesthetic drugs may be needed within the hospital system. 

A multidisciplinary team composed of anesthesiologists, the surgical team, and critical care clinical pharmacists could develop protocols to ensure conservation of sedatives used in the ICU and for surgical procedures. Agreements on the use of alternative agents must be considered for procedures being performed. Protocols addressing the level of sedation so as to avoid excessive sedation are an important consideration when making decisions. A Richmond Agitation Sedation Scale (RASS) assessment could be obtained in all intubated ICU patients at regular intervals. A score between −2 (light sedation) to 0 (alert and calm) is desirable [12]. Using the smallest effective dose to ensure that patients are comfortable while intubated will contribute to the conservation of medications. Deep sedation could be reserved for those that cannot tolerate lower doses. Practitioners could consider using alterative medications, such as fentanyl, benzodiazepines, ketamine, or dexmedetomidine. Our clinical pharmacist worked closely with the ICU doctors in recommending sedatives that were readily available. Utilization of medications with different routes of administration, other than intravenous administration, could also be explored. At our institution, clinical pharmacists developed recommended conversions for alternative medications. Many of our providers used this guide and switched to fentanyl patches for analgosedation. Oral medications or those that can be administered via a nasogastric or orogastric tube, or even rectally, are possible alternatives. Opioids and benzodiazepine medications can be administered via these routes. At our institution, clinical pharmacists developed an oral sedative dosing protocol for oral lorazepam. In addition, new paralysis order sets were developed for rocuronium and vecuronium. To address shortages of rocuronium and fentanyl, we procured these products by outsourcing with a 503b compounding pharmacy registered with the Food and Drug Administration (FDA) as an outsourcing facility [13]. Products compounded at these facilities may qualify for certain FDA exemptions with respect to labeling requirements but must abide by current good manufacturing practices (CGMP). It is imperative that research be conducted on the facility prior to any agreements to ensure that they fully comply with CGMP.

The backorder status of propofol was concerning at our institution due to a decrease in available stock. Although our stock was never depleted, our pharmacy team was proactive and discussed the possibility of procuring Propoven^®^ 2%. Although this product has not been approved by the FDA in the United States, it has been given EUA status and is considered an alternative to address propofol shortages for patients with COVID-19 requiring mechanical ventilation [14]. This double-strength propofol is a 20 mg/mL emulsion in 100 mL compared to the regular strength of 10 mg/mL and is indicated for ICU sedation only. The Institute of Safe Medication Practices (ISMP) recommends this medication should be thoroughly reviewed by a multidisciplinary team composed of critical care, nursing, and pharmacy staff prior to its use in the hospital [15]. Nursing and pharmacy staff may be unfamiliar with the product; therefore, procedures must be in place to avoid medication errors. This product has a higher concentration; therefore, nursing staff must be educated on its proper use to minimize dosing or intravenous pump errors, as there is great potential for overdoses to occur. The product should be stored separately from standard-strength propofol products to avoid mix-ups between the products, and warning stickers should be placed on the product to alert staff about its higher concentration. At our institution, we considered removing all propofol 1% products from the ICU stock to avoid confusion.

Practice/Learning Points: Shortages at the national and local levels may affect the availability of sedative medications. Working together as a multidisciplinary team will allow for alternative options to be explored to conserve much-needed medications so both patients undergoing surgery and those requiring mechanical ventilation can be comfortably sedated.

#### 3.1.5. Venous Thromboembolism Prophylaxis in Patients with Confirmed COVID-19 Infections

Case: The anticoagulation clinical pharmacist at our institution has been consulted to provide recommendations for venous thromboembolism prophylaxis in patients hospitalized due to COVID-19 infection. The consult specifically requested information regarding appropriate pharmacologic agents, doses, and duration of therapy, as well as non-pharmacologic strategies.

Answer: Prescribing practices for venous thromboembolism (VTE) prevention varied at our institution. Some practitioners used conventional doses of enoxaparin (a low-molecular weight heparin (LMWH)), while others used higher doses according to patient weight. Some practitioners also administered intravenous unfractionated heparin (UFH) for VTE prophylaxis. Therefore, it was extremely important for the clinical pharmacist to evaluate existing literature and to track VTE incidence in our inpatients with COVID-19. Although the latter task is still in progress, a review of the literature ensued to make recommendations for thromboprophylaxis in patients with COVID-19 and to identify whether SARS-CoV-2 contributes to hypercoagulability.

Infections due to various viral, bacterial, and fungal pathogens activate innate immunity, which triggers multifaceted systemic inflammatory responses. This leads to activation of coagulation factors and subsequent thrombin generation [16,17,18]. While SARS-CoV-2 does not appear to have intrinsic procoagulant effects, the resultant profound inflammatory response explains early reports describing abnormal coagulation parameters in patients with confirmed COVID-19 infection [19,20].

Clinical practice guidelines support the use of thromboprophylaxis in hospitalized patients with acute medical illness to reduce the risk of venous thromboembolism (VTE) [21,22,23]. Although a COVID-19-specific VTE risk assessment tool has not been established, several existing models account for complications and disease manifestation of COVID-19 (immobility, respiratory failure, and macro/microvascular diseases) and may be used. For example, the Padua model was used to stratify risk in a Chinese study in which 40% of hospitalized patients with COVID-19 were reported to have high risk of VTE [24].

In March 2020, the World Health Organization (WHO) published an interim guidance statement recommending the use of daily LMWHs, or twice-daily subcutaneous UFH at prophylactic doses [25]. The use of once-daily LMWH may be favored to reduce nursing exposure when administering therapy. The statement also suggested the use of intermittent pneumatic compression (IPC) when pharmacological prophylaxis is contraindicated. These suggestions were communicated with the Infectious Disease team and a decision was made to follow these strategies.

However, in April 2020, a consensus statement published by Zhai et al. highlighted the increased risk of VTE in patients with COVID-19 and strongly recommended the use of LMWH or UFH at the currently approved prophylactic doses [26]. However, the statement recommended adjusting the dose of LMWH according to patient weight, using higher doses in obesity. Additional recommendations suggested the use of twice-daily UFH in severe renal impairment. Similar to the WHO guidance statement, the use of IPC was suggested in the setting of contraindications to pharmacological therapy. The clinical pharmacist and Infectious Disease physician specialist discussed adjusting the dose in obese patients and decided to await more data before changing practice. This decision was mainly driven by an anecdotal report suggesting that the incidence of VTE in patients with COVID-19 is similar to patients without COVID-19.

In June 2020, The American College of Chest Physicians (CHEST) guidelines on the prevention, diagnosis, and treatment of VTE in patients with COVID-19 were published [27]. In critically ill patients with COVID-19, thromboprophylaxis with LMWH is recommended over UFH; however, both LMWH and UFH are recommended over fondaparinux and direct oral anticoagulants (DOACs). In acutely ill patients with COVID-19, thromboprophylaxis with LMWH or fondaparinux is recommended over UFH; however, all three agents are preferred over DOACs. Of note, due to limited evidence, the guidelines support the use of current standard prophylactic dosing over higher dosing strategies, such as weight-based dosing or twice-daily LMWH. Similar to previously published statements, the use of mechanical thromboprophylaxis is recommended when pharmacologic strategies are contraindicated. With regards to duration of the therapy, the CHEST guidelines do not support extended thromboprophylaxis beyond hospitalization. These guidelines reaffirmed the clinical pharmacist’s recommendations and allowed for a consistent prescribing practice with regards to thromboprophylaxis in patients with COVID-19.

Practice/Learning Point: Given the impact of COVID-19 infection on thromboembolic risk, it is imperative to continue to assess the ever-growing body of evidence as well as our own practice of VTE prevention in this patient population. At this time, while higher dosing and extended duration of VTE prophylaxis are not recommended, patient-specific characteristics and co-morbidities may inform a change in practice as more data are published.

## 4. Discussion

The COVID-19 pandemic has challenged healthcare providers and delivery systems in ways that were not planned for. It upended day-to-day activities and put strains on institutions to deliver care when treatment data were lacking, while maintaining measures to ensure the safety of patients and healthcare providers. As the old adage goes, ‘necessity is the mother of invention’ proved true when existing processes were modified in response to evolving evidence and needs. The cases presented in this commentary represent challenges that we encountered. Until the pandemic is controlled, learning about strategies from each other is important to allow resources to be allocated more efficiently. Through this series of real-life case scenarios, we hope to convey options that worked at our institution and that might be explored elsewhere. Furthermore, it opens the need to share more such innovative responses to challenges that a resource-constrained healthcare setting can adopt.
